# Harnessing Immune Cell Metabolism to Modulate Alloresponse in Transplantation

**DOI:** 10.3389/ti.2024.12330

**Published:** 2024-03-19

**Authors:** Johan Noble, Zuzana Macek Jilkova, Caroline Aspord, Paolo Malvezzi, Miguel Fribourg, Leonardo V. Riella, Paolo Cravedi

**Affiliations:** ^1^ Nephrology, Hemodialysis, Apheresis and Kidney Transplantation Department, University Hospital Grenoble, Grenoble, France; ^2^ Inserm U 1209, CNRS UMR 5309, Team Epigenetics, Immunity, Metabolism, Cell Signaling and Cancer, Institute for Advanced Biosciences Grenoble, University Grenoble Alpes, La Tronche, France; ^3^ Hepato-Gastroenterology and Digestive Oncology Department, University Hospital Grenoble, Grenoble, France; ^4^ Établissement Français du Sang Auvergne-Rhône-Alpes, R&D-Laboratory, Grenoble, France; ^5^ Translational Transplant Research Center, Icahn School of Medicine at Mount Sinai New York, New York, NY, United States; ^6^ Center for Transplantation Sciences, Department of Surgery, Massachusetts General Hospital, Harvard Medical School, Boston, MA, United States; ^7^ Division of Nephrology, Department of Medicine, Massachusetts General Hospital, Harvard Medical School, Boston, MA, United States

**Keywords:** solid organ transplantation, immune cells, metabolism, rejection, glycolysis

## Abstract

Immune cell metabolism plays a pivotal role in shaping and modulating immune responses. The metabolic state of immune cells influences their development, activation, differentiation, and overall function, impacting both innate and adaptive immunity. While glycolysis is crucial for activation and effector function of CD8 T cells, regulatory T cells mainly use oxidative phosphorylation and fatty acid oxidation, highlighting how different metabolic programs shape immune cells. Modification of cell metabolism may provide new therapeutic approaches to prevent rejection and avoid immunosuppressive toxicities. In particular, the distinct metabolic patterns of effector and suppressive cell subsets offer promising opportunities to target metabolic pathways that influence immune responses and graft outcomes. Herein, we review the main metabolic pathways used by immune cells, the techniques available to assay immune metabolism, and evidence supporting the possibility of shifting the immune response towards a tolerogenic profile by modifying energetic metabolism.

## Introduction

For many decades, transplant immunology has focused on the mechanisms of organ rejection and developing strategies to prevent graft injury by blocking key activation pathways in the recipient’s immune system [1]. Changes in the metabolism of the alloimmune cells have been regarded as the downstream effect of their effector function. More recently, it has become apparent that changes in immune cell metabolism can, by themselves, drive immune cell fate. The advent of novel technologies has allowed the collection of detailed data to decipher the plasticity of the metabolic state of immune cells [2]. These findings highlight the metabolic pathways in immune cells as a potential novel therapeutic approach to reprogramming immune responses and preventing transplant rejection [3].

Herein, we review the current knowledge on the importance of metabolic changes in immune responses, recent technologies to study immune metabolism, and how targeting immune cell metabolism could improve outcomes in SOT recipients.

## Cellular Metabolic Pathways in Immune Cells

Cellular metabolism is divided into anabolism and catabolism and both anabolic and catabolic reactions are essential for immune cell function and survival.

**FIGURE 1:**
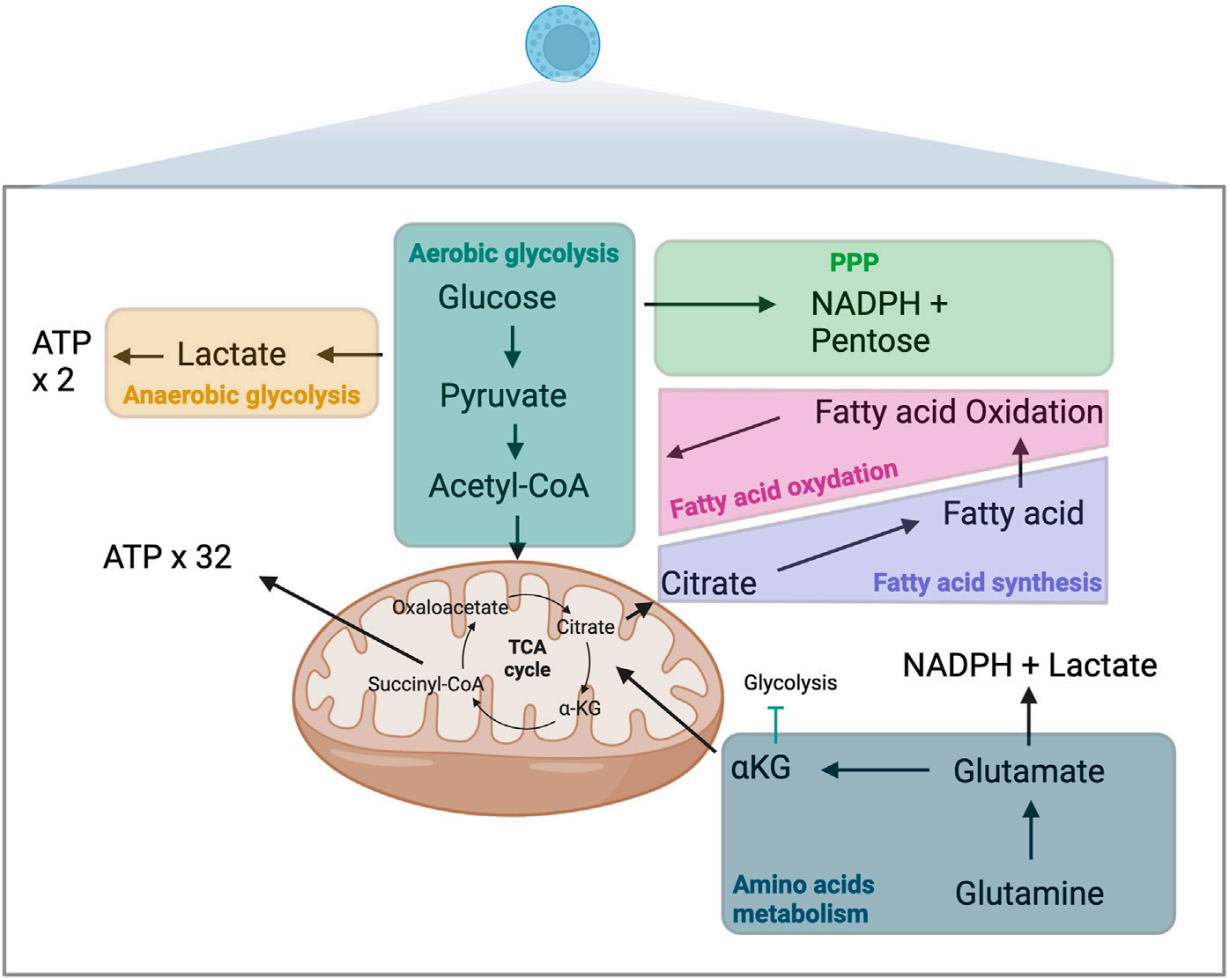
Metabolism and immune-metabolic pathways. ATP, adenosine triphosphate; αKG, α-ketoglutarate; TCA, tricarboxylic acid; NADPH, nicotinamide adenine dinucleotide phosphate; PPP, pentose phosphate pathway.

Anabolic reactions involve chain biosynthetic reactions that generates cell materials such as proteins and polypeptides from amino acids, DNA, RNA, lipids from fatty acid (FA). Anabolism require energy, typically provided in the form of adenosine triphosphate (ATP) molecules. Fatty acid synthesis (FAS) is a major anabolic reaction closely linked to immune cell function changes, differentiation, and proliferation [4]. Catabolic reactions involve the breakdown of complex molecules into simpler ones resulting in the release of energy such as proteins becoming amino acids or triglycerides breaking up into FA. Glycolysis and oxidative phosphorylation (OXPHOS) are the two main metabolic pathways that provide ATP for cells. Glycolysis refers to glucose oxidation to obtain ATP. OXPHOS refers to oxidation of nutriments within the mitochondria to generate ATP. Catabolic reactions are essential to support the high energetic requirements of immune cells, such as for cytokine production, rapid proliferation, and migratory activities (Figure 1).

### Sugars

Glycolysis, the breakdown of glucose, occurs in the cytosol of cells and is one of the primary catabolic processes contributing to the production of ATP [5]. The efficacy of the process depends not exclusively on oxygen availability and the mitochondrial capacity of immune cells. Aerobic glycolysis is the primary metabolic process contributing to energy generation in most immune cells. This highly efficient multi-step process starts with glucose molecule broken into two pyruvate molecules. In the presence of oxygen, which is required to re-oxidize nicotinamide adenine dinucleotide (NADH) to NAD^+^, pyruvate moves in the mitochondria and is converted to acetyl-CoA via pyruvate dehydrogenase. Acetyl-CoA enters in tricarboxylic acid (TCA) cycle and undergoes OXPHOS, leading to the production of 32 ATP molecules. In the absence of oxygen, glucose is metabolized in an anaerobic glycolysis process, through which pyruvate is converted into lactate, which yields only 2 ATP molecules. This process of lactate production can occur despite the presence of oxygen and fully functioning mitochondria (Warburg effect). Lactate also increases the NADH/NAD+ ratio. The pentose phosphate pathway (PPP) is an alternative pathway for glucose metabolism that generates nicotinamide adenine dinucleotide phosphate (NADPH) and pentoses (5-carbon sugars), essential moieties for synthesizing nucleotides. This pathway is crucial for effector functions of innate immune cells, including removing apoptotic cells including tolerogenic cells or activating and generating an oxidative burst of neutrophils [6, 7]. 5’ AMP-activated protein kinase (AMPK) is a metabolic sensor able to induce glycolysis trough the mammalian target of rapamycin complex (mTOR) pathway.

### Amino acids

Glutamine is an essential substrate for immune cell metabolism. This non-essential amino acid can be transformed into glutamate and then into α-ketoglutarate (αKG), which like glucose-derived acetyl-CoA, is an essential fuel for the TCA cycle [8]. The second fate of glutamine-derived glutamate involves its transformation into lactate and NADPH trough a truncated TCA cycle in which succinyl-CoA is converted into succinate, fumarate and then malate [9]. Outside the mitochondria, malate will be converted into pyruvate and then lactate. Amino acids other than glutamine have also been shown to play essential roles in immune metabolism [10]. Tryptophane derived metabolites such as kynurenine or kynurenic acid have also emerged as a major pathway involved in regulatory T cells generation, in auto-immune diseases and in tolerance [11].

### Fatty acids

Fatty acids (FAs) can fuel cellular metabolism through FA oxidation (FAO), another source of acetyl-CoA, which can then be shuttled to the TCA cycle. This metabolic pathway is of particular importance in adaptive immune responses [12]. AMPK is a metabolic sensor able to induce FAO.

## Techniques to Study Immune Cell Metabolism

Different methods to measure cell metabolism have been used [13]. Each technique represents a different approach and has advantages and limitations [14]. Table 1 summarizes the available tools to block or activate the different metabolic pathways.

**TABLE 1:** Drug targets that modify the metabolism of immune cells.

Metabolism pathway	Name	Targeted molecule	Effect on metabolism	Origin
OXPHOS	Oligomycin [15, 16]	ATP synthase	Inhibition	*Streptomyces* diastatochromogenes
OXPHOS	Rotenone [17]	Mitochondrial Complex I	Inhibition	Roots
OXPHOS	Antimycine A [18, 19]	Mitochondrial Complex III	Inhibition	*Streptomyces kitazawensis*
OXPHOS	Myxothiazol [20]	Mitochondrial Complex III	Inhibition	*Myxococcus fulvus*
OXPHOS and FAO	Metformin [21–23]	AMP Kinase Complex I FAO	Activation	*Galega officinalis*
			Inhibition	
			Increase	
Glycolysis	2-DG [24–26]	Hexokinase	nhibition	*De novo* synthesis
Glycolysis	Galactose [27, 28]	Pyruvate	Inhibition	Milk
Glycolysis	3-bromopyruvate [29]	Hexokinase II	Inhibition	*Escherichia coli*
Glycolysis	Ritonavir [30]	GLUT1 and 4	Inhibition	*De novo* synthesis
Glycolysis	FX11 [31]	LDHA	Inhibition	*De novo* synthesis
Glycolysis	DCA [32]	PDK2	Inhibition	*De novo* synthesis
Glycolysis	4-CIN [33]	Monocarboxylate transporter	Inhibition	*De novo* synthesis
Glycolysis	TEPP-46 [34–36]	PKM2	Inhibition	*De novo* synthesis
Glutamine	DON [37, 38]	Glutaminase	Inhibition	*Streptomyces*
Glutamine	BPTES [39]	Glutaminase	Inhibition	*De novo* synthesis
Glutamine	CK [40]	Glutaminase	Inhibition	*Escherichia coli*
FAO	Etomoxir [41, 42]	CPT1a	Inhibition	*De novo* synthesis
FAO	AICAR [43]	AMP kinase	Increase	*Escherichia coli*
FAS	C75 cerulenin, C75, orlistat, C93 [44]	Fatty acid synthase	Inhibition	*De novo* synthesis
FAS	TOFA [45]	Acetyl CoA carboxylase	Inhibition	*De novo* synthesis

AICAR, 5-aminoimidazole-4-carboxamide-1-beta-D-ribofuranoside; ATP, Adenosine triphosphate; BPTES, bis-2-(5-phenylacetamido-1,2,4-thiadiazol-2-yl)ethyl sulfide; CPT1a, Carnitine palmitoyl-transferase 1a; CK, L-2-amino-4-oxo-5-chloropentanoic acid; DON, 6-Diazo-5-oxo-L-Norleucine; DCA, dichloroacetic acid; FAO, Fatty acid oxidation; FAS, Fatty acid synthesis; FX11, 3-dihydroxy-6-methyl-7-(phenylmethyl)-4-propylnaphthalene-1-carboxylic acid; GLUT, Glucose transporter; PKM2, pyruvate kinase M2; LDHA, Lactate dehydrogenase-A; OXPHOS, Oxidative phosphorylation; PDK2, Pyruvate dehydrogenase kinase 2; TEPP, thieno-pyrrole-pyridazinone; TOFA, 5-tetradecyloxy-2-furoic acid; 2-DG, 2-deoxyglucose; 4-CIN, α-cyano-4-hydroxycinnate.

### Global Oxygen and Acidification Measurement

**FIGURE 2:**
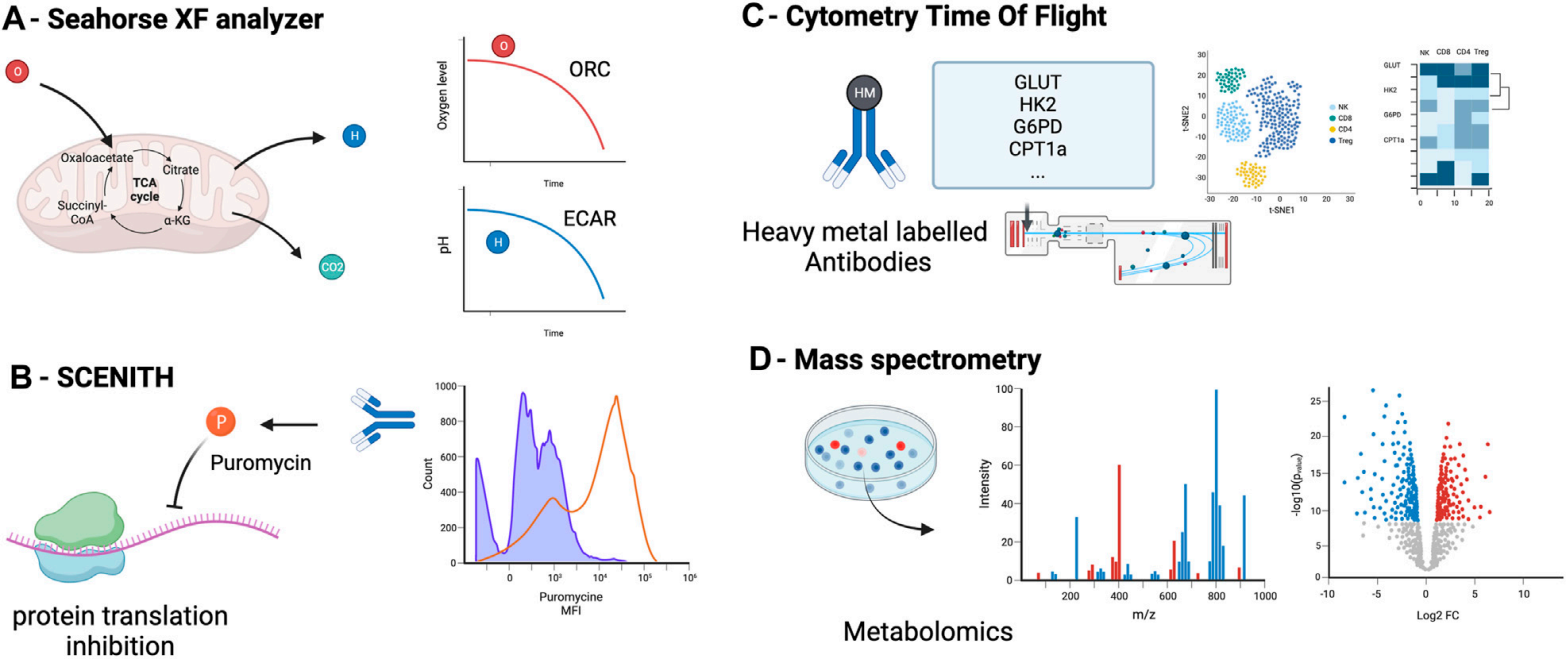
Principal methods to assess metabolism in immune cells: measurement of oxygen (OCR) and the extracellular acidification rate (ECAR) in the supernatant **(A)**, measurement of cell metabolism by single-cell energetic metabolism by profiling translation inhibition (SCENITH) **(B)**, Cytometry Time Of Flight **(C)** and metabolomics assessment by mass spectrometry **(D)** OCR, oxygen consumption rate, ECAR, extracellular acidification rate.

For the last decade, OXPHOS and glycolysis have been measured using the Seahorse ^®^ XF Analyzers (Figure 2A). This technique infers the oxygen consumption rate (OCR) through measuring the oxygen concentration in the supernatant of a cell culture over time, a surrogate marker of OXPHOS [46, 47]. Similarly, it estimates the extracellular acidification rate (ECAR) by measuring the changes in proton concentration in the supernatant over time, and using it as a surrogate marker of glycolysis [46]. OCR and ECAR can be assessed in parallel 96-well plate to perform replicates and multiple conditions.

This approach requires prior cell purification and it is unable to assess the metabolism at a single-cell level [48]. This method does not allow to perform simultaneous cell phenotyping nor cell sorting. Cells needs to be incubated from 12–18 h which can induce variability between wells in the number of cells and results.

### Single-Cell Energetic Metabolism by Profiling Translation Inhibition (SCENITH)

The SCENITH method has recently provided an interesting additional tool to assess immune metabolism at a single-cell level using flow cytometry [49]. This method assumes that most of the cell’s energy is employed for protein synthesis [50, 51]. By using the ability of puromycin to incorporate into protein during synthesis and our ability to detect it with an anti-puromycin antibody, the SCENITH method utilizes the level of puromycin incorporation as a marker of protein synthesis and thus, a surrogate marker of cell global metabolic activity (Figure 2B). The advantages of this technique include the possibility to study metabolism at a single cell level, the simultaneous study of multiple cell subtypes, the lack of sensitivity to metabolic modifications induced by the media, and the requirement of only a low number of cells (∼2000 cells) without purification. An additional advantage is the possibility of assessing cell phenotyping and other functions concomitantly [52]. The main limitation of the SCENITH method is its reliance on protein synthesis, which is only an indirect marker of cell metabolism, and lacks relevance for cells with low levels of protein synthesis, such as quiescent cells.

### Flow Cytometry and Cytometry by Time of Flight

By using a panel of key enzymes, flow cytometry can assess metabolic state. The “Met-Flow” panel includes 10 metabolic enzymes and transporters, including Hexokinase 1 for glycolysis, Carnitine palmitoyl-transferase 1A for FAO, Glucose-6-phosphate dehydrogenase [53]. This panel allows single-cell and phenotypic analysis of cell metabolism and does not require prior cell purification but the number of antibody needed may be a challenge.

Cytometry by time of flight (CyTOF) is another technique that can assess immune cell metabolism (Figure 2C). Instead of using fluorescent-labeled antibodies as in regular flow cytometry, cells are stained with antibodies conjugated to heavy metal isotopes [54], increasing the capacity to multiplex and reducing spectral overlap. About 110 metabolism-associated antibodies are available [55]. Recent studies reported a subset of 41–45 antibodies to target the important regulators (transporters, enzymes, signaling molecules, transcription factors) of metabolic pathways [56, 57]. CyTOF main limitation are the cost of the CyTOF equipment and the fact that this technique does not allow to recover living cells after analysis and thus only static measurement of single cells.

### Metabolomics

Metabolomics encompasses methods to detect and measure the cell metabolite levels and modifications (mass spectrometry combined with chromatography or ion mass, protein weight, ionization, and magnetic resonance) [58]. Carbon-labeled tracers can be added to mass spectrometry to specifically interrogate metabolic enzyme activities. Isotope tracers allow to quantify metabolomic flux on top of metabolic concentrations [59]. Mass spectrometry allow to detect and quantify even low concentration metabolites. Metabolites are detected according to their mass and charge. Classical separation techniques are liquid or gas chromatography. These methods require the least amount of material (about 200 cells without purification) and can target specific metabolites or all the metabolome (Figure 2D). Because of unbiased analysis, it also allows the discovery of new or uncharacterized metabolites. Limitations of this technique are the potential impact of the handling (medium and storage) on metabolite levels, the impossibility of combining with phenotypic analyses, the cost of the equipment and the variability resulting of the metabolites quenching and purification that can change rapidly the level of metabolites.

## How Metabolism Affects Immune Cells

Cellular metabolism does not only constitute a way to provide energy for immune cell survival and function, but it also regulates immune cell signaling pathways [60]. Metabolites have emerged as critical regulators of immune cell survival, differentiation, activation and function [5]. The metabolic network and its plasticity shape the fate and functions of both innate and adaptive immune cells [61].

### Innate Immune Cells

#### Myeloid Cells: Dendritic Cells and Macrophages

Activation of DC through Toll-like receptors (TLRs) is a crucial step for DC activation, maturation, as well as antigen processing and presentation. TLR engagement is associated with an increased level of glycolysis and a decreased level of OXPHOS [62–64]. Interestingly, DC can switch to OXPHOS metabolism when deprived of glucose due to competitive glucose uptake by T cells in the context of antigen presentation and T cell activation. Notably, glucose deprivation increases the capacity of DC to present and stimulate T cells [65].

An increase in glycolysis is critical in the initial phase of DC proliferation and differentiation, but then specific inflammatory or tolerogenic metabolic reprogramming follows [66]. This has been illustrated through SCENITH and CyTOF analyses of DC metabolism which show an increase of AMPK pathway and a decrease of mTOR pathway in tolerogenic DC as compared to inflammatory DCs [67]. Tolerogenic DC have been show to highly increase the ECAR level in the presence of glucose, to produce more lactate and have a higher lactate dehydrogenase activity as compared to other DC, suggesting a strong glycolytic profile of those cells [68].

In addition, many studies demonstrate that tumors promote specific metabolic pathways and nutriment uptake as compared to innate immune cells to reduce their effector functions and escape immuno-surveillance [69].

Different metabolic profiles characterize macrophage subsets. Pro-inflammatory macrophages (M1) have a higher succinate dehydrogenase in the TCA cycle which results in an increase of succinate which stabilize Hypoxia Inducible Factor 1 α (HIF1α) and in turns, promotes and sustain glycolysis activity [70]. Conversely, anti-inflammatory macrophages (M2) exhibit enhanced FAO and OXPHOS activity with an intact TCA cycle. Interestingly, increasing glutamine concentration *in vitro* culture medium drives mouse macrophage polarization into M2 profile, proving support to the notion that it is possible to orient the immune response through metabolism modifications [71]. αKG, a product of glutaminolysis, acts as a sensor of pro-anti-inflammation signals in mouse macrophages and can promote M2 polarization, but the role of glutamine in human macrophage is unknown [71].

#### Natural Killer Cells

The NK cell metabolic profile and effector functions depend on the context and the microenvironment. Cytokine-driven NK cell activation is associated with increased mitochondrial OXPHOS and glycolysis [72]. The relationship between metabolism and NK cell function was shown in tumor models, in which reduced availability of glucose and amino acids (leucine, arginine, glutamine) results in NK cell function impairment [73, 74]. Tryptophane pathway induction by indole 2,3-diamine oxygen (IDO) in tumors results in NK cell apoptosis to promote survival of cancer cells [75].

### T cells

#### Naive T cells

Metabolic program and T-cell activation are closely linked [8]. Before T Cell Receptor activation (TCR), naïve T cells are quiescent and have low ATP requirements. In their naïve state, their principal source of ATP is OXPHOS fueled by the oxidation of pyruvate and FAO, with a low glycolysis-based metabolism [8]. TCR engagement results in the activation, proliferation, and differentiation of the naïve T cells into effector, memory, and central memory T cells. This is paralleled by the transcription of key metabolic enzymes including the glucose transporter GLUT1 and the acetyl-CoA carboxylase 1 (ACC1) translation [76].

#### Activated T cells

T cell activation leads to major metabolic changes that favor glycolysis over OXPHOS [77].

Upon activation, CD8^+^ T cells undergo a first metabolic shift consisting of shuttling pyruvate to lactate metabolism [78], followed by a full switch from anaerobic to aerobic glycolysis [79]. The increase in glycolysis activity in activated CD8^+^ T cells is underpinned by an increase of glycolytic enzymes and an expression of glucose transporters such as GLUT1 [80]. Glycolysis inhibition results in cytokine and proliferation impairments in activated CD8^+^ T cells [81].

Effector CD4^+^ T cells, T helpers 1 (Th1), T helpers 2 (Th2), and T helpers 17 (Th17) cells are highly dependent on aerobic glycolysis, which is under HIF1α - mTOR regulation [82]. Although OXPHOS is more efficient in producing ATP, glycolysis gives the cells an advantage by rapidly providing the required energy for effector functions and proliferation. Interestingly, aerobic glycolysis is not needed for T cell activation, but it is strictly required for T effectors functions such as cytokine production (IL-2, IFN-γ mRNA translation and secretion) [77]. After TCR engagement and CD28 co-stimulation, the glucose uptake is increased by the upregulation of the cell surface glucose transporter GLUT-1 [83]. Interestingly, pharmacological blockade of glycolysis impairs Th1 and Th17 survival and function [84].

OXPHOS is also increased in activated CD4^+^ T cells even though the ratio OXPHOS/glycolysis is lower during T cell activation than in naïve CD4+T cells. The energy provided by OXPHOS seems to be mainly required during the first step of T cell activation, acting as an impulse [77]. CD4 T cells deficient for the mitochondrial complex III-derived Reactive oxygen Species (ROS) cannot activate and proliferate upon antigen presentation [85].

Amino acids are fundamental for activated T cells. Glutamine, a non-essential amino acid, constitutes an important energy source through glutaminolysis in activated T cells [86]. This is illustrated by the increase of glutamine transporters in activated T cells, and the reduced proliferation and cytokine secretion by T cells during glutamine starvation [87, 88]. Leucine have also been described to be of significant importance in the proliferation and differentiation of T cells trough [89].

T cell proliferation requires lipid synthesis to generate cell membranes for daughter cells. Blocking ACC1, a major enzyme for FAS, impairs Th1, Th2, and Th17 proliferation [90]. Interestingly, during Th17 cell development, but not regulatory T cells, FAS depends on ACC1, and blockade of this glycolytic-lipogenic pathway selectively impaired TH17 generation [90, 91].

#### Memory T cells

Memory T cells have a metabolic profile close to that of naïve T cells (lower glycolysis compared to OXPHOS) but with notable differences: they have a higher mitochondrial mass and a higher spare respiratory capacity which allow them to respond faster in case of antigen re-exposure [92]. Glycolysis in memory T cells is higher than in naïve T cells despite the similar ratio of glycolysis/OXPHOS [93]. As in regulatory T cells (Treg), FA constitute the principal fuel of OXPHOS for memory T cells [92]. FA come preferentially from *de novo* synthesis via the glycolytic-lipogenic pathway via mitochondrial citrate transformation and not from exogenous FA uptake, as in Tregs [94, 95].

AMPK is a regulator of FAO and glycolysis and the inability to generate memory T cells in AMPK-deficient mice is associated with deficient mitochondrial FAO [21]. Similarly, AMPK deficient CD8^+^ T cells are enable to generate memory CD8^+^ T cells [96–99]. In summary, metabolism signature of memory T cells remains uncertain while they exhibit an elevated profile of glycolysis and OXPHOS.

#### Exhausted T cells

In the context of persisting antigen and TCR stimulation, effector T cells progressively modify their phenotype to slowly become exhausted T cells, a low functional state phenotypically characterized by specific markers including programmed-death 1 (PD-1) [100]. In case of a high energy demand (glycolysis) sustained over time, glucose deprivation progressively drives a metabolic modification on effector T cells. These modifications are driven by the PD-1 pathway [101], resulting in a decrease in T cell glucose uptake and in OXPHOS [102], while FAO is upregulated [103].

#### Regulatory T cells

The metabolic profiles described above for other CD4^+^ T cells do not seem to apply to regulatory T cells (Treg), whose energy demands are not met through glycolysis but through OXPHOS and FAO [104].

Their independence from aerobic glycolysis has been shown *in vivo* in GLUT-1 deficient mice [105]. GLUT-1 deficiency was associated with impaired growth, proliferation, and survival of mature effector T cells, but did not affect either natural or induced Treg generation and expansion. The rate of glycolysis in Tregs is similar to that of naïve T cells but lower than in Th1 and Th17 cells [104, 105]. Inhibition of glycolysis using dichloroacetate increases Treg differentiation and promotes IL-10 production and FOXP3 expression [106]. Similarly, blocking glycolysis with 2-DG promotes Treg differentiation at the expense of Th17 [82].

In contrast, blocking OXPHOS results in Treg differentiation impairment [84]. In Treg, OXPHOS is fueled through FAO, blocking FAS has been shown to promote Treg generation, and FAO activity associates with an increase in AMPK activity. Adoptive transfer of modified OXPHOS or FAO deficient Tregs, resulted in a reduction of graft survival compared to wild-type [107]. Consistently, dysfunction of mitochondrial proteins (complex III, transcription factor A) is associated with Treg loss of function [108, 109].

### B cells

Activation of B cells trough B cell receptor (BRC) increases glucose and amino-acid uptake [110, 111]. However, glucose is not used for glycolysis, but for PPP and nucleotide synthesis [112]. OXPHOS and TCA cycle are augmented in activated B cells, but they are fueled by other source of energy than glucose, such as FAs [112, 113].

Following antigen activation, naïve germinal center B cells migrate into the follicle, where somatic hypermutation and antibody affinity maturation occur. During this process, B cells display a significant increase in OXPHOS activation [114].

Thereafter, B cells are transformed into short-lived plasma cells outside the lymphoid follicle and then into long-lived plasma cells and memory B cells inside the follicle. In plasma cells, the production of antibodies requires a high production of glutamate from glutamine pathway and a lower rate of glycolysis [115]. However, T-dependent long-lived plasma cells are characterized by a higher glucose and amino-acid uptake as compared to short-lived plasma cells [116].

## Immunometabolism in Solid Organ Transplantation

**FIGURE 3:**
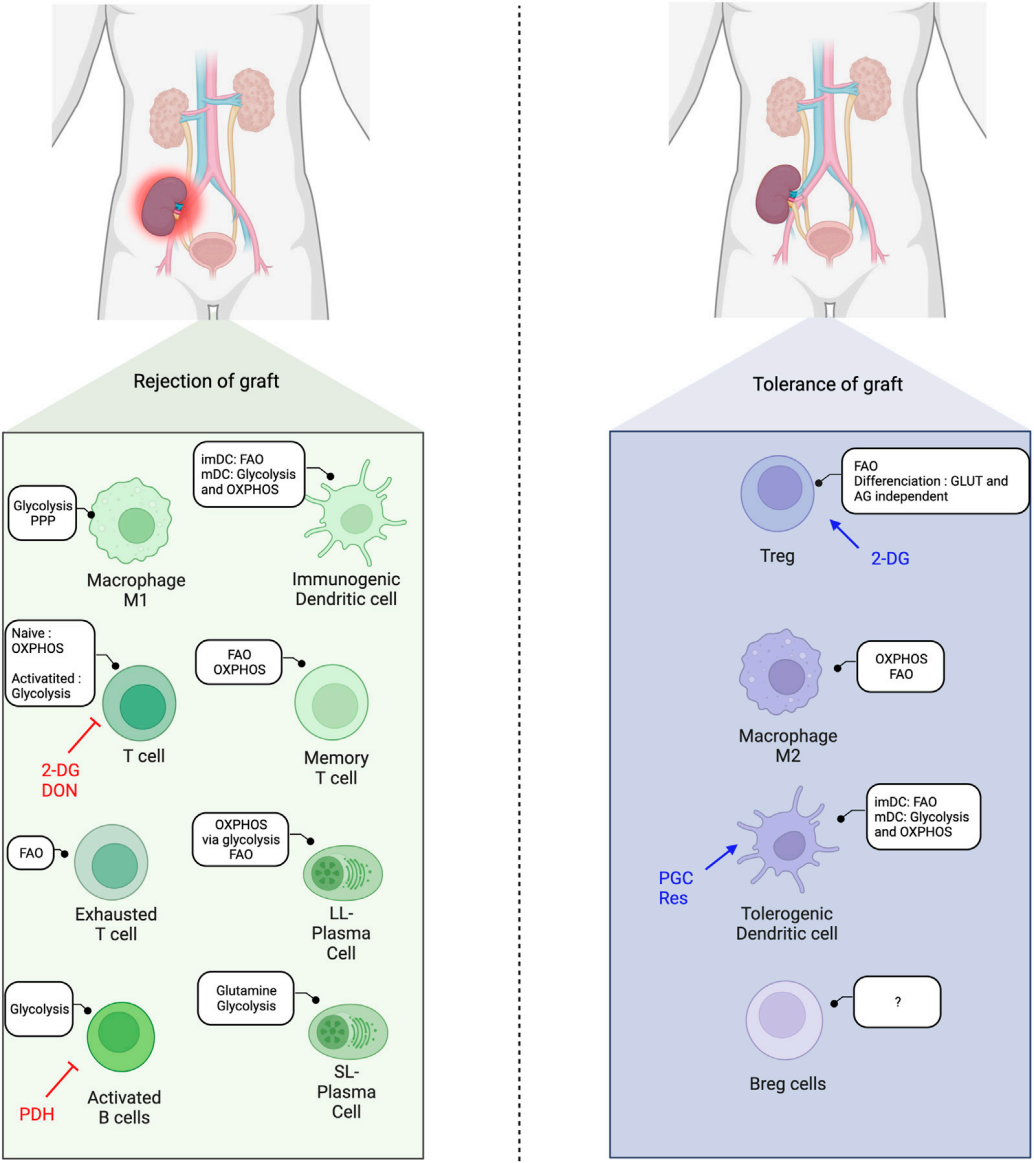
Immunometabolic balance in solid-organ transplantation and metabolic interventions. FAO, Fatty acid oxidation; OXPHOS, Oxidative phosphorylation; imDC, immature dendritic cells; mDC, mature dendritic cells; 2-DG, 2-deoxyglucose; DON, 6-Diazo-5-oxo-L-Norleucine; AG, aerobic glycolysis; Res, Resveatrol; PGC, PPARγ-coactivator-1β; PDH, pyruvate deshydrogenase; SL, short-lived; LL, long-lived.

As the metabolism impacts the development and function of immune cells, alterations in metabolic pathways can modulate immune cell differentiation and subsequently affect the balance between pro-inflammatory and regulatory cells, and thus influence transplantation outcomes (Figure 3).

### Modulation of Immune Metabolism in SOT

Few but very promising studies in murine transplant models highlight the impact of metabolic reprogramming on the alloimmune response [14].

In 2015, Lee *et al.* were able to modulate the alloimmune response in a fully mismatched murine model of skin and heart allograft transplantation by targeting metabolic pathways [117]. The authors showed that glycolysis inhibition using 2-DG and metformin hindered proliferation and cytokine production in activated T cells. Combination of 2-DG with the glutamine inhibitor 6‐diazo‐5‐oxo‐l‐norleucine (DON) resulted in an even more important inhibition of alloreactive CD4^+^ T cell proliferation and cytokine production, a decrease of acetyl-CoA levels and associated lipid synthesis, and a reduction of mTORC1 activation. The authors showed that in mice treated with 2-DG, metformin, and DON, CD4^+^ T cell kept their ability to differentiate into antigen-specific Foxp3+ CD4 T cells (Treg). Finally, the triple anti-metabolic therapy (2-DG, metformin, and DON), prolonged graft survival in a model of allogenic skin and heart transplantation, while discontinuation of treatment led to rapid graft rejection.

Immune metabolism may be modulated at the translational level. Quiescent CD4^+^ T cells accumulate a large amount of non-translated mRNA encoding key metabolic enzymes, which can be rapidly translated to activate aerobic glycolysis and FAO [76]. The engagement of the TCR triggers the translation of key proteins (GLUT1 and ACC1) for T cell activation and differentiation. Therefore, therapeutics that interfere with mRNA translation may affect alloimmunity by changing T cell metabolism.

Nian *et al.* showed that advanced age negatively impacts OXPHOS and glycolysis in naïve CD4^+^ T cells, and that glutaminolysis becomes the major source of ATP production later in life [118]. When the authors blocked OXPHOS with oligomycin, only CD4^+^ T cells from young mice were able to compensate for the metabolic loss with an increase in glycolysis. Interestingly, DON (glutaminolysis inhibitor) was able to inhibit IL-2, IFN-γ secretion, and cell proliferation in naïve CD4^+^ T cells from aged mice but not in naïve CD4^+^ T cells from young mice, which highlights the increasing reliance of naïve T cells on glutaminolysis with age. This approach prolonged graft survival and increased Tregs in a skin transplantation model in old mice. The results of DON on CD4^+^ T cells from aged mice were confirmed in human PBMC, suggesting its potential as a new age-dependent metabolic-mediated immunosuppression therapy [118].

### Immune Metabolism Modulation in Combination With Costimulation Blockade

In 2020, Lee *et al.* used the combination of their triple anti-metabolic therapy (metformin, 2DG and DON) in association with a co-stimulatory blocker (CTLA4-Ig) [119]. Their model showed that CTLA4-Ig and metabolic inhibition have distinct but synergic effects on immune cells. They first showed that metabolic inhibition resulted in a higher inhibition of proliferation and promotion of apoptosis than CTLA4-Ig. Interestingly, in a model of skin allograft acute rejection in mice, they also showed prolonged graft survival with anti-metabolic drugs compared to CTLA4-Ig and controls. This may be explained by the costimulation-independent activation of memory T cells that CTLA4-Ig did not block. When metabolic inhibitors were added to CTLA4-Ig, there was an additive inhibiting effect on T cell proliferation, T-bet expression, and cytokine secretion. Finally, CTLA4-Ig and metabolic inhibition were synergic in preventing skin and heart allograft loss in their mice transplantation model.

Moreover, CTLA4-Ig addition to metabolic inhibitors allowed long-term acceptance of heart allograft, which was not possible when anti-metabolic therapy was given alone [117]. Priyadharshini *et al.* proposed that a sequential with first metabolism blockade (2DG) associated with CTLA4-Ig may induce tolerance phase that could be maintained by adding secondarily mTOR inhibitors. This strategy could specifically increase the Treg and tolerogenic DC in the context of SOT [120].

## Conclusion

Recent studies and newly developed technologies have paved the way for modifying cell metabolism to influence the immune cell response. A better understanding of metabolic pathways in immune cells in the context of transplantation may offer the possibility to modulate the alloimmune response by reprogramming their metabolism to reshape specific immune cell subsets toward tolerogenic profiles. Further insights into metabolic dysregulation in SOT hold great promise to design novel therapies to improve graft and patient outcomes.

## Abbreviations

αKG, α-ketoglutarate; ACC1, acetyl-CoA carboxylase 1; AMPK, adenosine monophosphate–activated protein kinase; ATP, adenosine triphosphate; ECAR, extracellular acidification rate; FAO, fatty acid oxidation; HLA, human leukocyte antigen; IDO, indoleamine 2,3-dioxygenase; IFN, interferon; IL, interleukin; mTORC1, mammalian target of rapamycin complex 1; NADPH, nicotinamide adenine dinucleotide phosphate; OXPHOS, oxydative phosphorylation; OCR, oxygen consumption rate; PPP, pentose phosphate pathway; PBMC, peripheral blood mononuclear cells; SCENITH, single-cell energetic metabolism by profiling translation inhibition; SOT, solid-organ transplantation; TCA, tricarboxylic acid; TCR, T cell receptor; Tex, exhausted T cells; TLR, toll-like receptor; Treg, regulatory T cells.
